# *Mycoplasma hyopneumoniae* evades complement activation by binding to factor H via elongation factor thermo unstable (EF-Tu)

**DOI:** 10.1080/21505594.2020.1806664

**Published:** 2020-08-20

**Authors:** Yanfei Yu, Jia Wang, Rui Han, Li Wang, Lei Zhang, Amy Yimin Zhang, Jiuqing Xin, Shaoli Li, Yanhua Zeng, Guoqing Shao, Zhixin Feng, Qiyan Xiong

**Affiliations:** a Key Laboratory of Veterinary Biological Engineering and Technology of Ministry of Agriculture, National Center for Engineering Research of Veterinary Bioproducts, Institute of Veterinary Medicine, Jiangsu Academy of Agricultural Sciences, Nanjing, China; b School of Food and Biological Engineering, Jiangsu University, Zhenjiang, China; c College of Agriculture, Engineering & Science, University of KwaZulu-Natal, Durban, South Africa; d High Magnetic Field Laboratory, Chinese Academy of Sciences, Hefei, China; e College of Veterinary Medicine, Cornell University, Cornell, NY, USA; f Harbin Veterinary Research Institute, Chinese Academy of Agricultural Sciences, Harbin, China; g Department of Bacteriology, Capital Institute of Pediatrics, Beijing, China; h Institute of Pathogenic Biology, Hengyang Medical College, University of South China, Hengyang, China; i Institute of Life Sciences, Jiangsu University, Zhenjiang, China

**Keywords:** Mycoplasma, elongation factor Tu, complement factor H, complement activation, bacterial adhesion

## Abstract

Mycoplasmas persist in the host for a long time, suggesting that they possess mechanisms for immune evasion. Factor H is a negative regulator of the complement system, which binds to host cells to avoid unexpected complement activation. In this study, we revealed that many mycoplasmas, such as *Mycoplasma hyopneumoniae, Mycoplasma hyorhinis, Mycoplasma hyosynoviae, Mycoplasma gallisepticum, Mycoplasma pneumoniae, Mycoplasma genitalium, Mycoplasma flocculare*, and *Mycoplasma bovis* could hijack factor H such that they present themselves as a host tissue and thus escape from complement attack. Furthermore, the mechanism of recruiting factor H was identified in *M. hyopneumoniae. M. hyopneumoniae* binds factor H via factor H binding proteins, such as elongation factor thermo unstable (EF-Tu), P146, pyruvate dehydrogenase (acetyl-transferring) E1 component subunit alpha (PdhA), P46, Pyruvate dehydrogenase E1 component subunit beta (PdhB), glyceraldehyde-3-phosphate dehydrogenase (GAPDH), and three different hypothetical proteins. The binding of factor H by EF-Tu further contributes to decreased C3 deposition on the *M. hyopneumoniae* surface and ultimately blocks further complement activation. In fact, binding of factor H occurs in a multifactorial manner; factor H is not only exploited by *M. hyopneumoniae* via its regulator activity to help mycoplasmas escape from complement killing, but also increases *M. hyopneumoniae* adhesion to swine tracheal epithelial cells, partially through EF-Tu. Meanwhile, the high sequence identity among EF-Tu proteins in the above-mentioned mycoplasmas implied the universality of the mechanism. This is the first report that mycoplasmas can escape complement killing by binding to factor H.

## Introduction

Mycoplasmas, the smallest self-replicating bacteria, have low immunogenicity, and often persist in the host for a long time, suggesting that they possess mechanisms for immune evasion. For example, *Mycoplasma hyopneumoniae* can persist in host over several years [1], and has been cultured and isolated from the liver, spleen, kidneys, bronchial lymph nodes, and pericardial and synovial joint fluids of infected pigs [2–4]. It is not known how *M. hyopneumoniae* traffics to these sites, but it has been hypothesized that *M. hyopneumoniae* could hide from immunosurveillance, enter into various organs, and reside intracellularly [5]. As an essential part of innate immunity, especially in the bloodstream and body fluid, complement participates in host defense against infections, disposal of cellular debris, and inflammatory processes. Therefore, the present study attempted to reveal how mycoplasmas evade the complement system.

C3 convertase cleaves complement component C3 into C3b, which is the main effector molecule of the complement system that can covalently bind to the pathogen surface. This binding allows the innate recognition of microbes to be translated into effector responses. If no bond is made, C3b is inactivated, which is one way of inhibiting the alternative pathway in host cells [6]. Several regulators are necessary to control the processes that protect the host self-tissue from the potential injury caused by uncontrolled or misdirected complement activation. The plasma glycoprotein, factor H, is a key soluble inhibitor of complement activation in the alternative complement pathway that protects host cells from complement-mediated damage [7]. In addition to its regulatory activities in the fluid phase, it is believed that factor H also controls complement activation on cell surfaces [8].

Host cells are non-activators of complement and possess surface polyanionic molecules that permit factor H binding. Factor H binding to the cell surface can confer the ability to accelerate the intrinsic decay of C3/C5 convertases and/or act as a co-factor for the cleavage and inactivation of C3b by complement factor I [9]. The interactions of factor H with receptors on host cells thus protect host tissues from complement attack. By contrast, microbes are complement activators. Host-like sialic acids or glycosaminoglycans are not normally present on their surface. Therefore, microbes usually do not bind significant amounts of factor H, rendering them susceptible to complement attack [10].

However, many bacterial pathogens have evolved strategies to avoid complement activation, allowing them to escape elimination via this first line of innate defense. One mechanism is to express surface proteins that sequester soluble complement-regulatory proteins, such as factor H, to mimic host surfaces, thus protecting themselves from complement-mediated killing [10]. Some sialylated pathogens are able to recruit factor H because of the unusual presence of sialic acid on their surface, for example in *Neisseria gonorrhoeae* and *Pseudomonas aeruginosa* [11,12]. In addition to sialic acid-rich polysaccharide components, some surface-exposed proteins can also bind factor H. For example, the gram-negative pathogen *Neisseria meningitidis* produces a factor H-binding protein to recruit factor H to the pathogen membrane, permitting the pathogen to inactivate the C3b that is deposited on its surface, thereby avoiding the consequences of complement activation [13]. Non-typeable *Haemophilus influenzae* (NTHi) also binds to factor H [14], which is mediated by the cell-surface outer-membrane protein P5, contributing to the resistance of NTHi to complement killing [15,16]. The gram-positive pathogen *Streptococcus suis* binds factor H using two different surface proteins, Fhb and Fhbp, to degrade C3b in the presence of complement factor I [17,18]. *Streptococcus pneumoniae* and *Streptococcus pyogenes* also express factor H binding proteins [19,20]. By contrast, the inhibition of factor H-depleted sera as a complement source resulted in significantly increased C3 deposition on recombinant gp120-coated cells [21].

However, there has been little related research in mycoplasmas. In the present study, we aimed to evaluate the function of factor H in mycoplasmas and used *M. hyopneumoniae* as an example to identify the proteins involved in factor H binding. The role of the key proteins in mediating complement-dependent killing of *M. hyopneumoniae in vitro* is discussed. The findings are expected to facilitate the development of novel therapeutic interventions and improved therapies to treat mycoplasma-related diseases.

## Materials and methods

### Ethics statements

The authors confirm that the ethical policies of the journal, as noted on the journal’s author guidelines page, have been adhered to and the appropriate ethical review committee approved the study. The animal experiments were performed at Jiangsu Academy of Agricultural Sciences with the approval of the Committee on the Ethics of Animal Experiments (License No. SYXK (Su) 2015–0019). The procedures conformed to the guidelines of Jiangsu Province Animal Regulations (Government Decree No. 45), in accordance with the international law.

### Bacterial strains and growth conditions

The clonal isolates of the mycoplasmas were cultured at 37°C under conditions of stilling culture. The mycoplasmal pellets were harvested by centrifugation at 10,000 × *g* for 15 min at 8°C when the indicator in the culture medium changed color [22]. The specific information for the mycoplasmas used in this study is provided in Table 1.

**Table 1.** Survival of mycoplasmas preincubated with or without factor H in swine sera.

Mycoplasma species	Strain	Medium	^a^ Survival in sera preincubated with factor H	^b^ Survival in sera preincubated without factor H	Survival in complement-inactivated sera	Significance of difference of ^a^ and ^b^	Source
*M. hyopneumoniae*	168	KM2 + 15% swine sera	4.17E5 ± 5.05E4	3.98E3 ± 8.20E2	2.14E4 ± 4.61E3	**	Stored in our laboratory.
*M. hyosynoviae*	ATCC27095	ATCC243	1.00E4 ± 0	1.26E3 ± 2.59E2	1.42E3 ± 8.20E2	**	Purchased from ATCC.
*M. gallisepticum*	RH	KM2 + 15% swine sera	1.00E4 ± 0	1.00E3 ± 0	1.20E4 ± 2.60E3	**	From China Veterinary Culture Collection Center (CVCC).
*M. bovis*	PG45	PPLO+10% horse sera	3.98E5 ± 8.20E4	3.98E4 ± 8.20E3	4.80E4 ± 8.20E3	**	From Harbin Veterinary Research Institute, Chinese Academy of Agricultural Sciences.
*M. pneumoniae*	M129	K2 + 15% swine sera	1.00E4 ± 0	1.00E3 ± 0	2.64E4 ± 4.61E3	**	From Capital institute of Pediatrics.
*M. hyorhinis*	HF16	KM2 + 15% swine sera	2.24E5 ± 4.61E4	1.78E4 ± 0	2.70E4 ± 4.61E3	**	Stored in our laboratory.
*M. genitalium*	G73	KM2 + 15% swine sera	4.80E5 ± 8.20E4	3.98E4 ± 8.20E3	4.68E5 ± 8.20E4	**	From University of South China.
*M. mycoides* subsp. Capri	C87-1	KM2 + 15% swine sera	1.98E4 ± 6.32E3	1.78E4 ± 0	2.14 E4 ± 4.61E3	-	Purchased from CVCC.
*M. synoviae*	WVU1853	KM2 + 15% swine sera	3.16E5 ± 0	3.98E5 ± 8.20E4	3.80E5 ± 8.20E4	-	From The Poultry Disease Laboratory, Jiangsu Academy of Agricultural Sciences.
*M. flocculare*	ATCC27716	Friis+10% swine sera+10% horse sera	2.70E5 ± 4.6E4	3.52E4 ± 1.12E4	3.80E4 ± 8.20E3	**	Purchased from American Type Culture Collection (ATCC).

A p value ≤ 0.05 was considered highly significant“**”. “-” indicates no significant difference between the two treatments.

### Evaluation of the factor H binding activity of M. hyopneumoniae

To examine if *M. hyopneumoniae* cells could bind factor H, a cell-based enzyme-linked immunosorbent assay (ELISA) was performed. The 96-well microplates were sensitized using 100 μL of 5% glutaraldehyde at 37°C for 2 h. After washing three times with ultrapure water, each well was coated with 50 μL of bacterial suspension containing 10^8^, 10^7^, 10^6^, 10^5^, 10^4^ CCU (color change units) of mycoplasmas respectively, which was harvested and washed twice with phosphate-buffered saline (PBS) and inactivated with 0.8% formalin in advance. The wells were incubated overnight at 37°C for adsorption and drying. After blocking with 380 μL of 0.5% bovine serum albumin (BSA) in PBS-Tween 0.05% (PBST), 100 μL of human factor H (10 μg/mL) (Hycult Biotech, Uden, Netherlands) was added to the plates and incubation was carried out for 2 h at 37°C. Factor H bound to *M. hyopneumoniae* was detected by incubation with rabbit anti-factor H polyclonal antibodies (Abcam, Cambridge, MA, USA; 1:2000) and horseradish peroxidase (HRP)-conjugated goat anti-rabbit polyclonal antibodies (Boster, Pleasanton, CA, USA; 1:5000). Background values were obtained for the control (no factor H or wells coated with mycoplasma medium that was treated with the same method as the mycoplasma pellets). The OD450 value was recorded using a microplate reader after adding the HRP substrate. Each assay was performed in triplicate wells, and repeated independently three times.

### Complement killing mediated by factor H in M. hyopneumoniae and other mycoplasmas

The complement killing mediated by factor H in mycoplasmas, including *M. hyopneumoniae, M. pneumoniae, M. hyorhinis, M. hyosynoviae, M. gallisepticum, M. synoviae, M. genitalium, M. mycoides* subsp. Capri, *M. bovis*, and *M. flocculare* was evaluated. Fresh swine sera were used as the complement pool. Swine sera were collected from non-immunized crossbred (Xiaomeishan × Landrace) two-month-old piglets. All the swine sera samples used in this study were free of IgG antibodies against *M. hyopneumoniae, M. hyorhinis*, classical swine fever virus, porcine reproductive and respiratory syndrome virus, porcine pseudorabies virus (IDEXX Laboratories, Westbrook, ME, USA) and porcine circovirus type 2 (JBT, South Korea). The swine sera were also free of antigens of *M. hyopneumoniae* and *M. hyorhinis* [23]. Sera were sterilized by passing them through a 0.22-μm filter before use. A total of 5 × 10^5^ CCU of mycoplasma cells were washed twice with PBS and suspended in 50 μL of PBS. An equal volume of factor H (20 μg/mL in PBS) or PBS was added into the above mycoplasma suspensions and preincubated at 37°C for 30 min. Then, 400 μL of normal swine sera was added into the mixture and incubated at 37°C for 2 h. The reaction mixtures were then placed on ice and the survival of bacteria in the presence of sera was determined using a CCU assay. Three samples were assayed in each experiment. Differences were analyzed using Student’s t- test [24].

### Identification of factor H binding proteins of M. hyopneumoniae by two-dimensional electrophoresis (2-DE) and far-western blotting analysis

#### Surface proteins extraction

*M. hyopneumoniae* cells at the mid-log phase of growth were washed with PBS and suspended in 1% (wt/vol) Triton X-114 in PBS. The mixture was incubated on ice for 2 h and centrifuged at 13,000 × *g* for 30 min at 4°C. The supernatant was removed and incubated at 37°C for 5 min for rapid condensation of Triton X-114 and then centrifuged at 13,000 × *g* for 10 min at room temperature. The lower Triton phase was freed from the aqueous phase by extraction another two times. The final Triton phase was precipitated using ethanol. The obtained protein precipitate was purified using a two-dimensional (2-D) Clean-up Kit (GE Healthcare, Chicago, IL, USA)

### 2.5.2 2-DE analysis of membrane proteins

The 2-DE and far-western blotting analysis were performed according to Li’s protocol, with slight modifications [25]. In brief, two aliquots of the precipitated proteins were resuspended in 250 µL of rehydration solution (7 M urea, 2 M thiourea, 2% w/v CHAPS (3-((3-cholamidopropyl) dimethylammonio)-1-propanesulfonate), 0.2% w/v DTT (dithiothreitol), 0.5% v/v IPG buffer (an ampholyte-containing buffer), 0.002% w/v bromophenol blue), and incubated at 25°C for 30 min. The soluble supernatants were loaded onto a 13 cm immobilized pH gradient (IPG) gel strip (Immobiline DryStrip, pH 3–10; Bio-rad, Hercules, CA, USA) for isoelectric focusing (IEF). The samples were rehydrated at 30 V, 20°C for 12 h, and then with stepwise increases in the voltage as follows: 500 V for 4 h, 1,000 V for 1 h, 2,000 V for 1 h, 4,000 V for 1 h, 8,000 V for 2 h, and 8,000 V for 1.5 h (max. voltage, 8,000 V; max. current, 50 µA/IPG strip; total 28,000 V h). The strips were then subjected to 12% sodium dodecyl sulfate polyacrylamide gel electrophoresis (SDS-PAGE) in the second-dimension. One gel was stained with Coomassie brilliant blue (CBB-G250) for mass spectrometry analysis, while the duplicate gel was transferred onto a polyvinylidene fluoride (PVDF) membrane for subsequent far-western blotting analysis. Three replicates were run for each sample.

#### Far-western blotting analysis

The PVDF membrane was blocked overnight at 4°C with 5% (w/v) skimmed milk diluted with TBST containing 0.05% Tween 20, 50 mM Tris-HCl buffer (pH 7.4), and 150 mM NaCl. The membrane was washed three times and incubated with 10 μg/mL of factor H dissolved in TBST overnight at 4°C. Following three washes with TBST, the membranes were incubated with rabbit anti-factor H antibodies (Abcam; 1:2000) at 37°C for 2 h. HRP-conjugated goat anti-rabbit polyclonal antibodies (Boster; 1:5000) was used to detect the factor H binding proteins. The positive spots were developed with 3,3ʹ-diaminobenzidine (DAB; Tiangen, Beijing, China). The images scanned from the PVDF membrane and the corresponding 2-DE gels were analyzed using Imagemaster 7.0 (GE Healthcare). The two images were aligned using the layer function of Adobe Photoshop [26].

#### MALDI-TOF MS and database searches

Spots that corresponded to those identified as immunoreactive were excised from the 2-DE Coomassie brilliant blue G250-stained gel and analyzed using matrix-assisted laser desorption/ionization-time of flight-mass spectrometry (MALDI-TOF-MS)/MALDI-TOF-TOF-MS following in-gel tryptic digestion (Steed BioTechnologies Co. Ltd., Nanjing, China). Peptide mass fingerprinting (PMF) data were analyzed using the Mascot server (http://www.matrixscience.com), a software used to analyze mass spectroscopy data. Peptides with a Mascot rank of 1 in the Mascot search were considered significant and used for the combined peptide score.

### Screening for key factor H binding proteins

To investigate the contribution of the identified factor H binding proteins to infection by *M. hyopneumoniae*, the virulence factors reported in previous publications were taken into consideration for the analysis [27]. The STRING database (https://string-db.org/) was used to generate protein-protein interactions between the factor H binding proteins identified in this study and known virulence factors. Only interactions with a confidence score of at least 0.4 were considered for analysis. The protein-protein interaction network was visualized using Cytoscape (3.8.0).

### Factor H binding ability of M. hyopneumoniae elongation factor thermo unstable (EF-Tu)

To further demonstrate the observed EF-Tu – factor H interaction as a specific binding with physiological significance for *M. hyopneumoniae*, an antibody blocking assay was performed [28]. Briefly, 10 µg of factor H in carbonate buffer was used to coat wells of 96-well microplates, which were incubated at 4°C overnight. Unbound factor H was washed away using PBST three times and the wells were blocked using 5% BSA. A total of 10^6^ CCU of *M. hyopneumoniae* cells, which had been preincubated with heat-inactivated specific polyclonal antibody against recombinant EF-Tu (1:2000) or preimmune serum, were added separately to the wells and incubated for 1 h at 37°C. The plates were washed three times to remove unbound *M. hyopneumoniae* and treated with lysis buffer containing 0.025% (v/v) Triton X-100 and 0.1% trypsin at 37°C for 10 min, followed by quantitation using quantitative real-time polymerase chain reaction (qPCR) [29]. The percentage adhesion of mycoplasmas incubated with factor H was calculated from the ratio of copies recovered from cells incubated with factor H to those incubated with normal sera, and is reported as the fold change of adhesion along with the standard error of the mean. The background values were obtained for the control (no *M. hyopneumoniae* cell treatment). Each assay was performed in triplicate wells, and repeated independently three times. All data are expressed as mean ± the standard error of the mean (SEM). Data were analyzed for significance using one-way analysis of variance (ANOVA), followed by Student’s t-test for comparisons between two groups. A p value ≤ 0.05 was considered significant (**).

### Different recombinant domains of EF-Tu, and the preparation of polyclonal antibodies

EF-Tu comprises three independent domains. To identify which region is responsible for factor H binding, the *tuf* gene from *M. hyopneumoniae* was divided into three parts encoding the three different domains, which were cloned into pET-32a separately using homologous recombination technology (ClonExpress®-One Step Cloning Kit, Vazyme Biotech Co., Ltd, Nanjing, China). The sequence that overlapped with the end of the cloning site was added onto the insert using PCR. Table 2 lists the primers used in this study. The reconstructed plasmids were transformed into *Escherichia. coli* BL21 (DE3) for isopropyl-β-d-thiogalactopyranoside (IPTG)-inducible expression, and the induced proteins were purified using Ni-chelating chromatography (GenScript, Nanjing, China).

**Table 2.** Primers for recombinant expression of *ef-tu* with a restriction site of *Bam*H I and *Xho* I based on homologous recombination technology.

Primer Name	Summary of Functions or Sequences (5′- 3′)
EF-Tu-IF	GCCATGGCTGATATCGGATCCCATATTAATATTGGAACAATT
EF-Tu-IR	GTGGTGGTGGTGCTCGAGTTA AGGTGAGTCAATATAAGAATC
EF-Tu-IIF	GCCATGGCTGATATCGGATCCTTTTTGATGGCCGTTGAGGAT
EF-Tu-IIR	GTGGTGGTGGTGCTCGAGTTATGGTTTTGCAATAACCTGACC
EF-Tu-IIIF	GCCATGGCTGATATCGGATCCCCGCATACAAAATTTAAAGCT
EF-Tu-IIIIR	GTGGTGGTGGTGCTCGAGTTAAACTGTTCCGGCACCCACGGT

Polyclonal antibodies against the recombinant proteins were prepared by subcutaneously immunizing 1-month-old New Zealand white rabbits. Each rabbit was immunized three times with 1 mg of purified recombinant proteins emulsified in Freund’s adjuvant (Sigma, St. Louis, MO, USA) at 2-week intervals. Sera were collected one week after the third immunization. The use of animals in this study met the legal and ethical requirements, and the animals were treated humanely. Antibodies against the full-length EF-Tu were prepared in our previous study [29].

### ELISA binding assay of the different domains of EF-Tu with factor H

To examine the binding ability of the different regions of EF-Tu to immobilized factor H and to further verify the findings of far-western blotting analysis, 96-well microplates were coated with 100 μL of 10 μg/mL of the different regions of EF-Tu at 4°C overnight. After blocking with 380 μL of 0.5% BSA in PBST, a series of concentrations of factor H (0, 0.2, 1, 5, and 10 μg/mL) in 100 μL were added to the plates and incubation carried out for 2 h at 37°C. The wells were then washed three times with PBST and incubated with rabbit anti-factor H polyclonal antibodies (Abcam; 1:2000) at 37°C for 1 h. After three washes, HRP-conjugated goat anti-rabbit polyclonal antibodies (Boster; 1:5000) were added and incubated for another 1 h. Binding was measured after adding the substrate 3,3,5,5-tetramethylbenzidine and the absorbance of plates were read using an ELISA microplate reader at 450 nm. Background values were obtained for the control (coated with 10 μg/mL casein). Each assay was performed in triplicate wells, and repeated independently three times.

### Surface plasmon resonance analysis of EF-Tu and factor H

The interaction dynamics of full-length EF-Tu and factor H were further investigated in real time using surface plasmon resonance (SPR) in a BIAcore X100 Plus instrument (GE Healthcare). Factor H was diluted to 10 µg/mL in 10 mM sodium acetate (pH 4.0) and covalently linked to the carboxylmethylated dextran matrix of a CM5 sensor chip as a ligand using an amine coupling kit (Biacore AB, Uppsala, Sweden). Immobilization of soluble factor H generated 712 resonance units (RU). Binding kinetics were measured using increasing concentrations (0–14 μM) of the analyte (recombinant EF-Tu) in running buffer (HBS-EP) consisting of 10 mM HEPES, 150 mM NaCl, 3 mM EDTA, and 0.05% (v/v) surfactant P20 (Biacore AB) at a flow rate of 30 μL/min for 180 s over immobilized factor H at 20°C. The dissociation phase was monitored for 1000 s by allowing the buffer to flow over the chip. Association kinetics were analyzed manually using Biacore X100 Control Software [30].

### Detection of surface C3 deposition on M. hyopneumoniae using flow cytometry

Flow cytometry analysis was used to detect if the binding of factor H by EF-Tu further influenced complement deposition on the surface of *M. hyopneumoniae*. In brief, fixative *M. hyopneumoniae* strain 168 (10^8^ CCU) was incubated with anti-EF-Tu primary serum at a 1:100 dilution at 37°C for 1 h (preimmune rabbit serum was used as the negative control). After washing with PBS, the cells were incubated with or without 10 μg/mL of factor H at 37°C for 1 h. The cells were then incubated with 10 μg/mL of complement C3 component (ImmunoClone, Huntington Station, NY, USA) at 37°C for 1 h. To detect the deposition of complement, the cells were incubated with fluorescein isothiocyanate (FITC)-conjugated anti-C3 antibody (Santa Cruz Biotechnology, Santa Cruz, CA, USA). Finally, the fluorescence intensity was detected using a flow cytometer (FACSJazz, BD Biosciences, San Jose, CA, USA) [31].

### Adherence of M. hyopneumoniae to swine tracheal epithelial cells (STECs) mediated by factor H

To investigate the ability of factor H to promote *M. hyopneumoniae* adherence to STECs, STECs were grown to confluence in 24-well plates with Roswell Park Memorial Institute (RPMI)-1640 medium (Thermo Fisher Scientific) supplemented with 10% (v/v) fetal bovine serum (Gibco, Grand Island, NY, USA). *M. hyopneumoniae* cells of 10^7^ CCU were incubated with or without 10 μg of factor H, washed three times with PBS, and then incubated with STECs. The STECs were preincubated with or without anti-factor H antibodies (1:20) before incubation with *M. hyopneumoniae*. Following incubation, the wells were washed three times with PBS to remove unbound *M. hyopneumoniae* cells. Then, the cells were treated with lysis buffer containing 0.1% trypsin and 0.025% (v/v) Triton X-100, followed by bacterial genome extraction and qPCR for bacteria counting [32]. The assay was performed in triplicate, and the data were analyzed using Student’s *t* test using SPSS 20.0 (IBM Corp., Armonk, NY, USA). For all tests, a value of p ≤ 0.05 was considered statistically significant.

### Immunohistochemistry and EF-Tu sequence aligment

To detect the distribution of factor H in *M. hyopneumoniae* colonization sites, the bronchia of *M. hyopneumoniae*-infected pigs collected from the experiment performed in a previous study were subjected to immunohistochemical analysis [33]. The bronchial tissues were fixed by incubating with 100 mL of 4% polyformaldehyde for 24 h at room temperature. They were then placed into histology cassettes and embedded in paraffin. Four-µm-thick sections were cut for bronchial transection and processed for immunohistochemical staining. To stain the sections for factor H, the slides were blocked with 5% BSA and incubated with a 1:100 dilution of anti-factor H antibodies at 4°C in a humidified chamber. To stain the sections for *M. hyopneumoniae*, the slides were blocked with 5% BSA and incubated with a 1:100 dilution of anti-P97 monoclonal antibodies at 4°C in a humidified chamber. The negative control slides were treated identically except that PBS was used instead of the primary antibody. The slides were washed three times with PBS between each incubation. Bound antibodies were detected using HRP-conjugated antibody and a chromagen solution containing 3-amino-9-ethylcarbazole and 0.015% hydrogen peroxide in dimethylformamide. The slides were counterstained with hematoxylin. The slides were examined under a Leica microscope [34].

The amino acid sequence alignment of EF-Tu proteins from *M. hyopneumoniae, M. hyorhinis, M. hyosynoviae, M. gallisepticum, M. pneumoniae, M. genitalium, M. flocculare*, and *M. bovis* was peformed using the Multiple Sequence Alignment function of DNAMAN software version 9.

## Results

### *Factor H* protects M. hyopneumoniae and seven other mycoplasmas *from complement activation*

**Figure 1.**
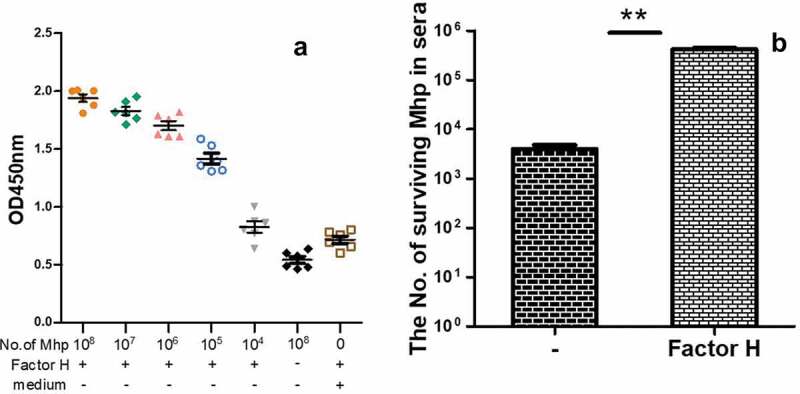
The factor H binding ability of *M. hyopneumoniae* strain 168 and its influence on *M. hyopneumoniae* survival in swine sera.

Previous studies demonstrated that various bacteria could interact with factor H to evade complement killing [35,36,36], while no research was reported in mycoplasmas. Therefore, we used *M. hyopneumoniae* as an example to determine whether mycoplasmas interact with factor H. The binding of factor H to the cell surface of *M. hyopneumoniae* was detected using a cell-based ELISA binding assay. As shown in Figure 1(a), compared with the no factor H wells or the medium-coated wells, the interaction of *M. hyopneumoniae* and factor H was significant. Thus, *M. hyopneumoniae* is able to bind factor H to its surface. Increased numbers mycoplasmas could bind more factor H. The ability of factor H binding is proportional to the number of mycoplasma cells. To further explore the consequences of the interaction for *M. hyopneumoniae*, the survival of mycoplasmas in the complement pool (the swine sera), incubated with and without factor H, was calculated. The results are shown as CCU with the SEM. *M. hyopneumoniae* preincubated with factor H was resistant to complement killing (83.4% survival), while those without factor H were readily killed (0.8% survival) (Figure 1(b)). The binding of Factor H produced significant protection against complement killing for *M. hyopneumoniae*. To explore if the mechanism was universally significant to more mycoplasmas, the complement killing mediated by factor H in other mycoplasmas was also evaluated. The data listed in Table 1 show that, with the exception of *M. mycoides* subsp. Capri and *M. synoviae*, all the other seven tested mycoplasmas, including *M. hyorhinis, M. hyosynoviae, M. gallisepticum, M. pneumoniae, M. genitalium, M. flocculare*, and *M. bovis*, showed significantly increased survival in the complement pool after incubation with factor H. These data demonstrated that factor H might play an important role in the protection of *M. hyopneumoniae* against complement killing. The mechanism was significant in other mycoplasmas, including *M. hyorhinis, M. hyosynoviae, M. gallisepticum, M. pneumoniae, M. genitalium, M. flocculare*, and *M. bovis*.

(a) The factor H binding ability of *M. hyopneumoniae* strain 168 incubated with rabbit anti-factor H polyclonal antibodies and horseradish peroxidase (HRP)-conjugated goat anti-rabbit polyclonal antibodies. Background values were obtained for the control (no factor H or wells coated with *M. hyopneumoniae* culture medium). The OD450 was used for the statistical analysis. Each assay was performed in triplicate wells, and repeated independently three times(b) The survival of *M. hyopneumoniae* cells in the presence of normal swine sera (used as a complement pool) as determined by assaying for CCU (color change unit) after incubating with an equal volume of factor H or PBS. Three samples were assayed in each experiment.Differences were analyzed using the Student’s t- test. A p value ≤ 0.05 was considered highly significant (**). Mhp is short for *M. hyopneumoniae*.

### Identification of M. hyopneumoniae factor H binding proteins using proteomics, far-western blotting, and protein-protein interaction analysis

**Figure 2.**
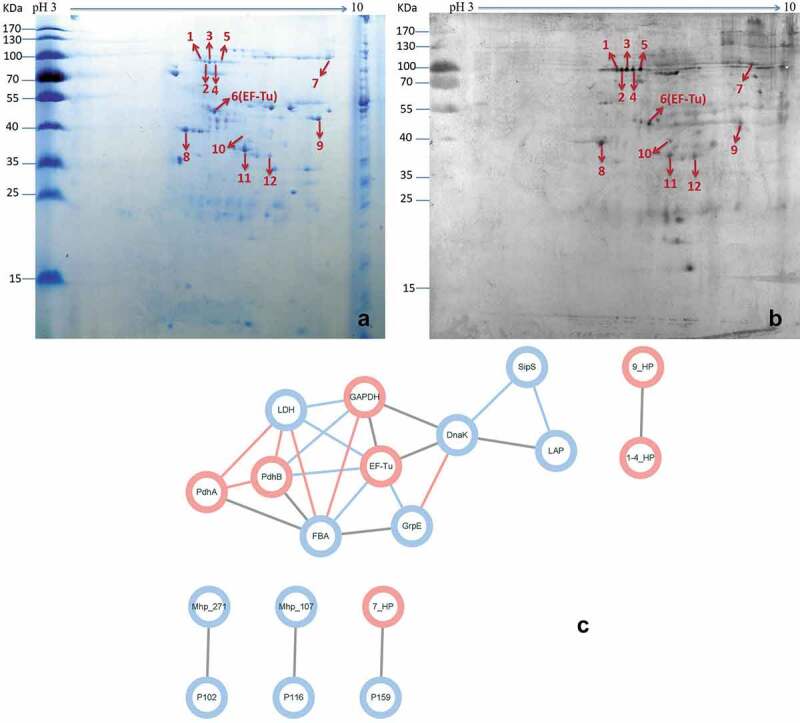
Identification of important factor H binding proteins of *M. hyopneumoniae.*

To identify which surface proteins are responsible for the interaction between *M. hyopneumoniae* and factor H, a method combining 2-DE proteomics and far-western blotting analysis was developed in this study. Figure 2(a) shows the membrane proteins of *M. hyopneumoniae* displayed on the 2-DE gel. Figure 2(b) shows the proteins spots showing reactivity with factor H after transfer to a PVDF membrane. According to their position on the PVDF membrane, the corresponding reactive spots were excised from the repeated 2-DE gel and subjected to MALDI-TOF-MS/MALDI-TOF–TOF-MS analysis, followed by peptide mass fingerprinting (PMF) searching. Twelve potential factor H-binding peptides were successfully identified, which belonged to nine different *M. hyopneumoniae* surface proteins, respectively. Information for the identified spots is provided in Table 3. This analysis showed that the *M. hyopneumoniae* proteins EF-Tu, P146, PdhA, P46, PdhB, GAPDH, and three different hypothetical proteins are responsible for binding to factor H. More importantly, EF-Tu showed the highest score in the Mascot search results, which indicated it as the most promising candidate. The protein-protein interaction network further demonstrated the hub position of EF-Tu among the factor H binding proteins during *M. hyopneumoniae* infection. A total of 25 direct physical interactions among the factor H binding proteins identified in this study and known virulence factors are shown in Figure 2(c). Seven factor H binding proteins were implicated in the interaction network. The factor H binding proteins (pink nodes) are strongly linked to each other, as well as to the known virulence factors (blue nodes). EF-Tu showed the most links to other proteins among the factor H binding proteins (Data Sheet 1). EF-Tu connected virulence factors and factor H binding proteins, and formed an important hub protein in the network. The results indicated that these seven factor H binding proteins are involved in *M. hyopneumoniae* virulence, and EF-Tu was one of the most important proteins in the network. Additionally, EF-Tu has been identified as one of the most important factor H binding proteins in other bacteria [35], and is a verified key adhesin of *M. hyopneumoniae* [29,33]. Therefore, EF-Tu was selected as a representative potential factor H-binding protein for further study in *M. hyopneumoniae*.

**Table 3.** Summary of the analysis performed on the protein spots identified using MALDI-TOF-MS/MALDI-TOF-TOF-MS.

Spot no.^a^	Protein description	Protein ID	Mass	Top Score of Mascot Search Results^b^	Matches^c^	Sequences
1	hypothetical protein	WP_014580031.1	145720	77	1(1)	1(1)
2	hypothetical protein	WP_014580031.1	145720	216	3(3)	3(3)
3	hypothetical protein	WP_014580031.1	145720	252	3(3)	3(3)
4	hypothetical protein	WP_014580031.1	145720	145	2(2)	2(2)
5	P146 adhesin like-protein	AAZ08057.1	147533	74	1(1)	1(1)
6	elongation factor Tu (EF-Tu)	WP_011206373.1	44153	266	7(6)	7(6)
7	hypothetical protein	WP_014579912.1	215057	159	2(2)	2(2)
8	pyruvate dehydrogenase (acetyl-transferring) E1 component subunit alpha (PdhA)	WP_011206102.1	42355	78	1(1)	1(1)
9	hypothetical protein	BAB17620.1	26345	66	1(1)	1(1)
10	46 kDa surface antigen (P46)	P46_MYCH2	45431	19	1(1)	1(1)
11	Pyruvate dehydrogenase E1 component subunit beta (PdhB)	WP_011206101.1	36768	157	2(2)	2(2)
12	type I glyceraldehyde-3-phosphate dehydrogenase (GAPDH)	WP_011205875.1	37098	88	1(1)	1(1)

^a^Protein spots corresponding to the position on the gel and blot (Figure 1(a,b)).

^b^The threshold of significance was greater than 95% (p < 0.05) for all values in this study.

^c^Data in parentheses indicate the sequence coverage factor.

(a) Coomassie G-250-stained two-dimensional gel of surface proteins of *M. hyopneumoniae* strain 168 cultured in KM2 medium (pH 3–10, 13 cm). The numbers on the left indicate the molecular weights of the standards (kDa); the numbers in the profile represent different independent spots.(b) Far-western blot of the corresponding gels of surface proteins from *M. hyopneumoniae* strain 168 using factor H and anti-factor H antibodies. The numbers on the left indicate the molecular weights of the standards (kDa); the numbers in the profile represent different reactive spots that can bind factor H.(c) Interaction networks of the factor H binding proteins and known virulence factors. Protein-protein interactions derived from String database with a confidence score ≥ 0.4 are displayed. The blue edges show interactions of medium confidence (0.4), gray edges indicate high confidence (0.7), and the confidence (0.9) is displayed as pink edges. Blue nodes represent the known putative virulence factors collected from published papers and pink nodes represent the factor H binding proteins identified in this study. Blue lines represent interactions between two nodes.

### EF-Tu is important for M. hyopneumoniae binding to factor H

**Figure 3.**
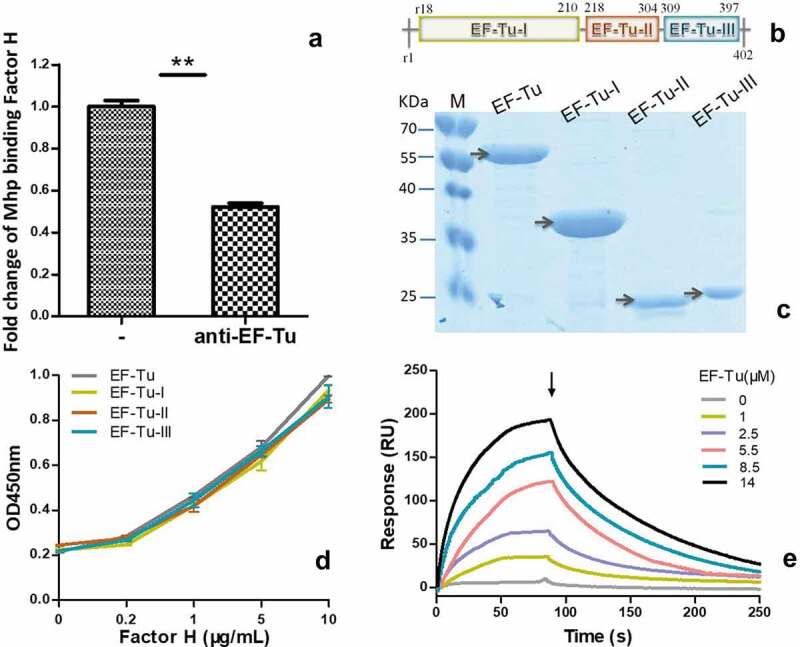
The interaction of *M. hyopneumoniae* elongation factor thermo unstable (EF-Tu) with factor H.

*M. hyopneumoniae* strain 168 produces EF-Tu, one of the surface proteins that potentially bind to factor H. To investigate the contribution of EF-Tu to factor H binding in *M. hyopneumoniae*, an ELISA test was performed. The percentage binding of *M. hyopneumoniae* incubated with anti-EF-Tu antibodies was calculated from the ratio of copies binding factor H from cells incubated with anti-EF-Tu antibodies to those incubated with normal sera. As shown in Figure 3(a), after incubation with anti-EF-Tu antibodies, the amount of *M. hyopneumoniae* binding to factor H immobilized on 96-well plates decreased significantly. EF-Tu contributed 47.7% of *M. hyopneumoniae*’s binding ability to factor H. Thus, EF-Tu is important for *M. hyopneumoniae’*s binding to factor H.

(a) The percentage of adhesive *M. hyopneumoniae* incubated with factor H was calculated from the ratio of copies recovered from cells incubated with anti-EF-Tu antibodies to those incubated with normal sera, and is illustrated as the fold-change of adhesion along with the standard error of the mean (SEM). Data were analyzed for significance using the one-way analysis of variance (ANOVA) test followed by a Student’s t-test for comparisons between two groups. A p value ≤ 0.05 was considered highly significant (**). Mhp is short for *M. hyopneumoniae*.(b) Diagram of the three independent domains of EF-Tu.(c) The different recombinant domains purified by nickel affinity chromatography. The first lane (M) comprises protein markers. The second to fifth lanes are the full-length recombinant EF-Tu, the first domain, the second domain, and the third domain of EF-Tu respectively.(d) Binding of different concentrations of factor H to recombinant EF-Tu detected using microplates coated different domains of EF-Tu. The OD450 was obtained using the addition of anti-factor H antibodies and horseradish peroxidase (HRP)-conjugated goat anti-rabbit polyclonal antibodies. Data are expressed as the mean ± standard error of the mean (SEM) of three independent experiments performed in triplicate.(e) Sensorgrams depicting the binding of immobilized factor H to recombinant full-length EF-Tu. Increasing concentrations of EF-Tu (0, 1, 2.5, 5.5, 8.5, and 14 μM) were injected at a flow rate of 30 μL/min for 180 s over immobilized factor H. The arrow indicates the end of the injection period, at which point dissociation of EF-Tu from factor H cold be observed. RU = resonance units.

### Each domain of EF-Tu contributes equally to the interaction with factor H

Recombinant full-length EF-Tu and its three independent domains were prepared and purified (Figure 3(b–c)). Corresponding polyclonal antibodies were also prepared. To further evaluate the key binding sites of EF-Tu to factor H, ELISA was performed. As shown in Figure 3(d), all the different regions of recombinant EF-Tu, including domain I, II, and III, and full length EF-Tu, could interact with factor H in a concentration-dependent manner. These results confirmed the far-western blotting analysis, in which the captured EF-Tu of *M. hyopneumoniae* interacted specifically with factor H. However, the whole protein only displayed a slightly higher OD, which was not significantly different from the other groups. There were no significant differences in the interactions of the different regions of EF-Tu with factor H. The key binding sites may be distributed widely along the full protein. Therefore, in this study, we chose full-length EF-Tu as a representative to further measure of real-time interactions between EF-Tu and factor H using surface plasmon resonance (SPR) (Figure 3(e)). The results were consistent with those expected for a specific, moderately strong interaction between proteins of these sizes, and EF-Tu was found to bind factor H in a dose-dependent and physiologically relevant manner, with a *K*_D_ = 7.825 × 10^−6^ M.

### C3 deposition increased on M. hyopneumoniae after binding to factor H using EF-Tu for resistance to direct complement-dependent lysis

**Figure 4.**
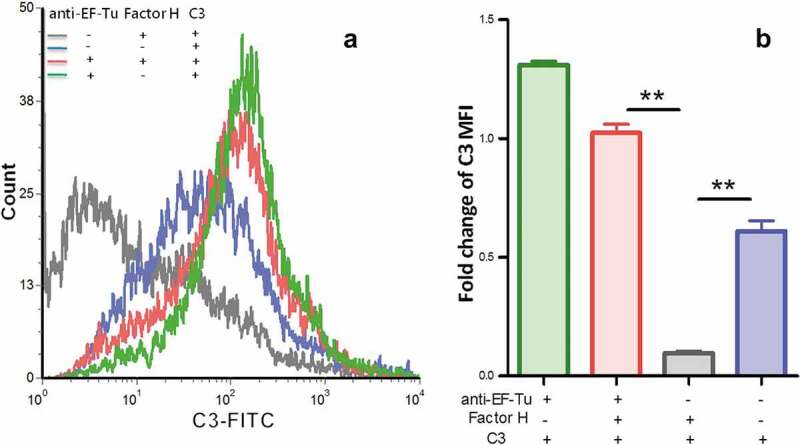
Detection of C3 deposition on the surface of *M. hyopneumoniae* using flow cytometry.

Complement-dependent killing might contribute to clearance of *M. hyopneumoniae*. We sought to determine whether *M. hyopneumoniae* resisted direct killing by complement (mediated by insertion of the membrane attack complex [C5b-9]) by resisting the deposition of C3. *M. hyopneumoniae* cells were preincubated either with EF-Tu antibody or preimmune sera; with or without the addition of factor H to the wash mixture; and C3 fragments deposited on mycoplasmas were measured using flow cytometry. As shown in Figure 4, the addition of anti-EF-Tu antibodies markedly increased C3 fragment deposition on the surface of mycoplasmas, which was increased by about eight-fold above the levels observed with the addition of preimmune sera. Without EF-Tu, no significant decrease of C3 on *M. hyopneumoniae* was observed. Adding factor H markedly decreased C3 fragment deposition on the surface of mycoplasmas, which decreased by about five-fold below the levels seen without the addition of factor H. Without factor H, the decrease of C3 on *M. hyopneumoniae* was also not significant. These data suggested that both EF-Tu and factor H are important for *M. hyopneumoniae*’s ability to decrease C3 deposition. *M. hyopneumoniae* might recruit factor H through EF-Tu. As a negative regulator of complement activation, the deposition of factor H on *M. hyopneumoniae* further blocked the central component of complement system, C3 deposition. Without EF-Tu or factor H, the influence on *M. hyopneumoniae* C3 deposition markedly reduced. The blockade of complement function following C3 deposition might ultimately result in resistance of *M. hyopneumoniae* to direct complement-dependent killing, which would help the survival of *M. hyopneumoniae* in swine sera.

(a) The gray line indicates *M. hyopneumoniae* strain 168 successively treated with preimmune serum, factor H, C3 protein, and fluorescein isothiocyanate (FITC)-conjugated C3 antibody; the blue line indicates *M. hyopneumoniae* strain 168 treated with preimmune serum, C3 protein, and FITC-conjugated C3 antibody; the red line indicates *M. hyopneumoniae* strain 168 treated with anti- EF-Tu serum, factor H, C3 protein, and FITC-conjugated C3 antibody; the green line indicates *M. hyopneumoniae* strain 168 treated with anti-EF-Tu serum, C3 protein, and FITC-conjugated C3 antibody.(b) The mean fluorescence intensity (MFI) of C3 deposition in *M. hyopneumoniae* incubated without anti-EF-Tu sera or factor H (the blue, gray, and green columns) is expressed as the fold-change of the corresponding strain incubated with anti-EF-Tu serum, factor H, C3 protein, and FITC-conjugated C3 antibody (the red column). The asterisks above the charts indicate statistically significant differences.

### The influence of factor H on adherence

**Figure 5.**
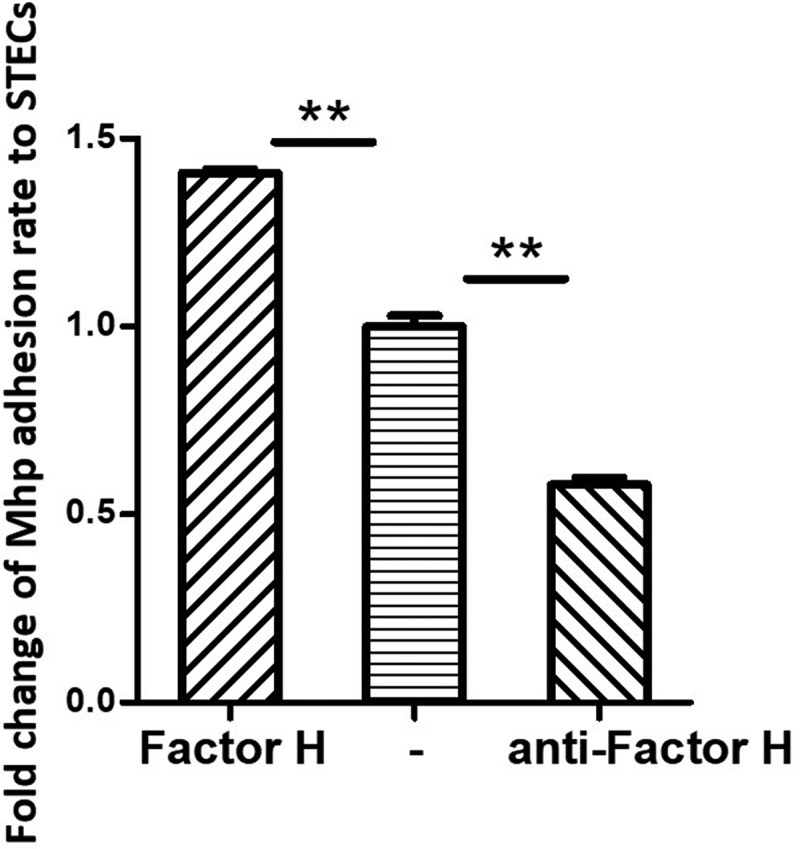
*M. hyopneumoniae* Adhesion influenced by factor H.

An adherence assay was used to further assess the contribution of factor H to the adhesion of *M. hyopneumoniae*. Factor H was incubated with *M. hyopneumoniae* cells before adherence to STECs. The level of adherence of *M. hyopneumoniae* incubated with factor H was expressed as the percentage of *M. hyopneumoniae* incubated without factor H. The results revealed that after incubation with factor H, the adherence efficiency of *M. hyopneumoniae* to STECs increased by 41% (P < 0.05) compared with those incubated with BSA (Figure 5). These results confirmed that factor H could increase the adherence of *M. hyopneumoniae* to host cells. Besides, the antibody inhibition assay was used to further assess the contribution of factor H to the adhesion of *M. hyopneumoniae*. Anti-factor H antibodies were incubated with STECs before incubation with *M. hyopneumoniae* cells. The level of adherence of *M. hyopneumoniae* to STECs incubated with anti-factor H antibodies was expressed as the percentage of *M. hyopneumoniae* adherence to STECs incubated without anti-factor H antibodies. The results revealed that after incubation with anti-factor H antibodies, the adherence efficiency of *M. hyopneumoniae* to STECs was reduced by 43% (P < 0.05) (Figure 5). These results further confirm that factor H plays an important role in the adherence of *M. hyopneumoniae* to host cells.

Left column: Fold-change of the factor H adhesion rate: The number of bacteria recovered in the cells incubated with factor H/number of bacteria recovered in the group incubated without factor H (middle column) × 100%. Data are expressed as means ± SD of at least three experiments with samples assessed in triplicate.Right column: Fold-change of the adhesion rate: The number of bacteria recovered from the swine tracheal epithelia cells (STECs) incubated with anti-factor H antibodies/number of bacteria recovered from the group incubated without factor H antibodies (middle column)× 100%. Data are expressed as means ± SD of at least three experiments with samples assessed in triplicate. Mhp is short for *M. hyopneumoniae*; STECs is short for swine tracheal epithelial cells.

### Abundant factor H is expressed in the M. hyopneumoniae colonization site

**Figure 6.**
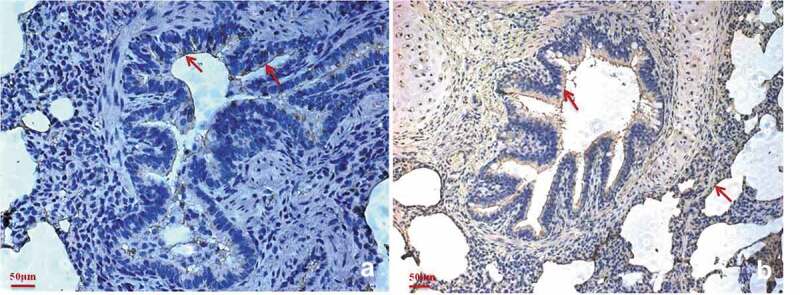
Factor H is predominantly present in the *M. hyopneumoniae* colonization site.

Immunohistochemical staining was performed to explore whether factor H is distributed in *M. hyopneumoniae* colonization sites *in vivo*. Bronchiole sections obtained from *M. hyopneumoniae*-challenged pigs were subjected to immunohistochemical staining. The samples stained positive for factor H, especially along the ciliary borders of the bronchioles (Figure 6(a)). The bronchioles also stained positive for P97, which is one of the main surface proteins of *M. hyopneumoniae*. The ciliary borders and alveoli are the preferred colonization sites of *M. hyopneumoniae*, as shown in Figure 6(b). The result showed that factor H is available at the colonization site along the airways, especially in the ciliary borders of the bronchioles, which is the preferred location for *M. hyopneumoniae* colonization. This would provide an environment for *M. hyopneumoniae* to utilize factor H *in vivo* for complement evasion and adherence. Thus, the interaction of *M. hyopneumoniae* and factor H is of physiological significance in the host.

Immunohistochemical staining of porcine bronchiole sections from *M. hyopneumoniae*-challenged pigs was performed using anti-factor H antibodies (a) or anti-P97 monoclonal antibodies (b). The presence of red staining shows the location of factor H (a) or *M. hyopneumoniae* strains (b). A widespread distribution of factor H can be observed in the bronchiole sections of all samples, especially in the ciliary borders of the bronchioles that are colocalized by *M. hyopneumoniae*.

### EF-Tu might play a wide role in more mycoplasmas

**Figure 7.**
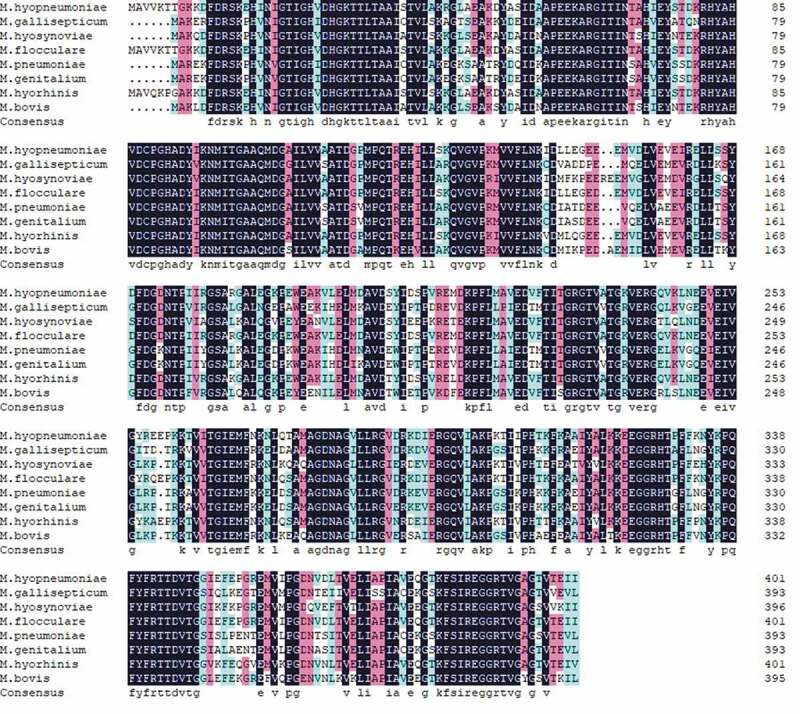
The sequence alignment of EF-Tu proteins from different mycoplasmas.

To further predict the function of EF-Tu in other mycoplasmas that showed factor H-mediated complement evasion and adherence, a multiple alignment of the amino acid sequences of EF-Tu proteins from *M. hyopneumoniae, M. hyorhinis, M. gallisepticum, M. pneumoniae, M. genitalium, M. flocculare, M. bovis*, and *M. hyosynoviae* was performed. As shown in Figure 7, the homology among the EF-Tu amino acid sequence of the eight mycoplasmas is 83.23%, which indicated that EF-Tu is an important factor H binding protein not only in *M. hyopneumoniae*, but also in *M. hyorhinis, M. gallisepticum, M. pneumoniae, M. genitalium, M. flocculare, M. bovis*, and *M. hyosynoviae*. Therefore, EF-Tu might play a wide role in mycoplasmas.

The amino acid sequence alignment of EF-Tu proteins from different mycoplasmas that showed factor H-mediated complement evasion, including *M. hyopneumoniae, M. hyorhinis, M. hyosynoviae, M. gallisepticum, M. pneumoniae, M. genitalium, M. flocculare*, and *M. bovis* was illustrated using DNAMAN. The color of the frame indicates the identity of the amino acid sequence. The darker the color, the higher the sequence identity.

## Discussion

The specific physiological interactions of factor H with host cells and ligands allow recognition of self and non-self to protect the host from the damage caused by complement activation. One way in which factor H functions as a negative regulator of complement activation is by acting as a cofactor in the proteolysis of C3b, which can be covalently linked to the host surface to trigger the subsequent response and formation of the membrane attack complex [37]. The important targets for factor H binding on host cells are host-specific polyanionic surface molecules, such as glycosaminoglycans and sialic acid, which are not displayed frequently by microbes [38]. However, many pathogens have adapted to avoid complement-mediated killing by hijacking factor H to their surface using non-host ligands [39]. For example, *N. gonorrhoeae* [40], *Haemophilus influenzae* [31], and *Moraxella catarrhalis* [41] could bind to factor H via their surface-exposed factor H binding proteins.

In this study, we reported that *M. hyopneumoniae* could bind factor H. Nine different *M. hyopneumoniae* membrane proteins were identified as being responsible for factor H binding; EF-Tu, P146, PdhA, P46, PdhB, GAPDH, and three hypothetical proteins. Among the nine proteins that might bind to factor H, we focused on EF-Tu, the candidate with highest score in the MS analysis and showed that it formed a hub position among the factor H binding proteins. The kinetic curve of the binding reaction of EF-Tu to factor H was further evaluated *in vitro*, which showed a moderately strong affinity. Further physiological significance was explored using *M. hyopneumoniae* strains. Antibodies recognizing EF-Tu could block factor H binding in *M. hyopneumoniae*, confirming EF-Tu’s importance as a factor H binding protein. After recruiting factor H to the mycoplasmal surface using EF-Tu and some other factor H binding proteins, the quantity of C3 deposition was significantly reduced, which suggested inhibition of complement activation, demonstrating that *M. hyopneumoniae* has developed mechanisms to recruit factor H to its surface to decrease C3 deposition and eventually avoid complement attack. In addition, we showed that factor H was abundant along the ciliary borders of pig bronchioles, where *M. hyopneumoniae* most commonly colonizes the host. This provides superior conditions for factor H function *in vivo*. Furthermore, the mechanism of binding to factor H to escape from the complement killing was confirmed to exist widely in many mycoplasmas, such as *M. hyorhinis, M. hyosynoviae, M. gallisepticum, M. pneumoniae, M. genitalium, M. flocculare*, and *M. bovis*.

Recruitment of factor H to the bacterial surface is multifactorial. In addition to its complement regulatory function, factor H can mediate functions unrelated to this regulatory activity. Recruitment of factor H might also help bacteria to adhere and to invade epithelial and endothelial cells [42–44]. The results obtained in this study also showed an important role of host factor H in the process of *M. hyopneumoniae* adhesion to epithelial cells. Binding of factor H to the *M. hyopneumoniae* surface increased its adhesion to epithelial cells significantly and vice versa, and anti-factor H antibodies blocked the binding. This observation suggested that this mechanism could mainly benefit bacteria during the early stages of infection. The observation that pathogens bind factor H regions distinct from its complement inhibitory domains has been used to engineer factor H fusion proteins lacking complement-inhibitory activity to the Fc region of IgG as a therapeutic target to direct Fc-mediated clearance [45]. This approach would be attractive to treat infections with *M. hyopneumoniae* and other mycoplasmas, and provides direction for further research. However, both pathogenic and nonpathogenic microbes demonstrate the ability to bind to factor H to help microorganisms evade complement activation, which shows that it does not necessarily play a decisive role in pathogenic microbe immune escape [10]. In addition, avoiding all complement-mediated killing might only occur after blocking multiple complement regulatory proteins, such as decay accelerating factor, factor H, membrane cofactor protein, and CD59, because these proteins could have an additive effect in which one regulatory protein could compensate for the loss of another.

In summary, we showed that binding of factor H to the surface *M. hyopneumoniae* by EF-Tu occurs in a multifactorial manner. It not only assists *M. hyopneumoniae* escape from complement killing by decreasing C3 deposition, but also increases *M. hyopneumoniae* adhesion to epithelial cells. The mechanism of binding to factor H to escape from the complement killing exists widely in many mycoplasmas, such as *M. hyorhinis, M. hyosynoviae, M. gallisepticum, M. pneumoniae, M. genitalium, M. flocculare*, and *M. bovis*. Furthermore, the amino acid sequence of the factor H binding protein EF-Tu in these mycopalsmas showed high homology, which indicated that EF-Tu might play an important and extensive role in factor H binding in mycoplasmas.

## Supplementary Material

Supplemental Material
