# Supplementary figures and images for: Mycoplasma hyopneumoniae evades complement activation by binding to factor H via elongation factor thermo unstable (EF-Tu)
Source: Virulence. 2020 Aug 20;11(1):1059–74. doi: 10.1080/21505594.2020.1806664 (PMC7549910; doi:10.1080/21505594.2020.1806664)

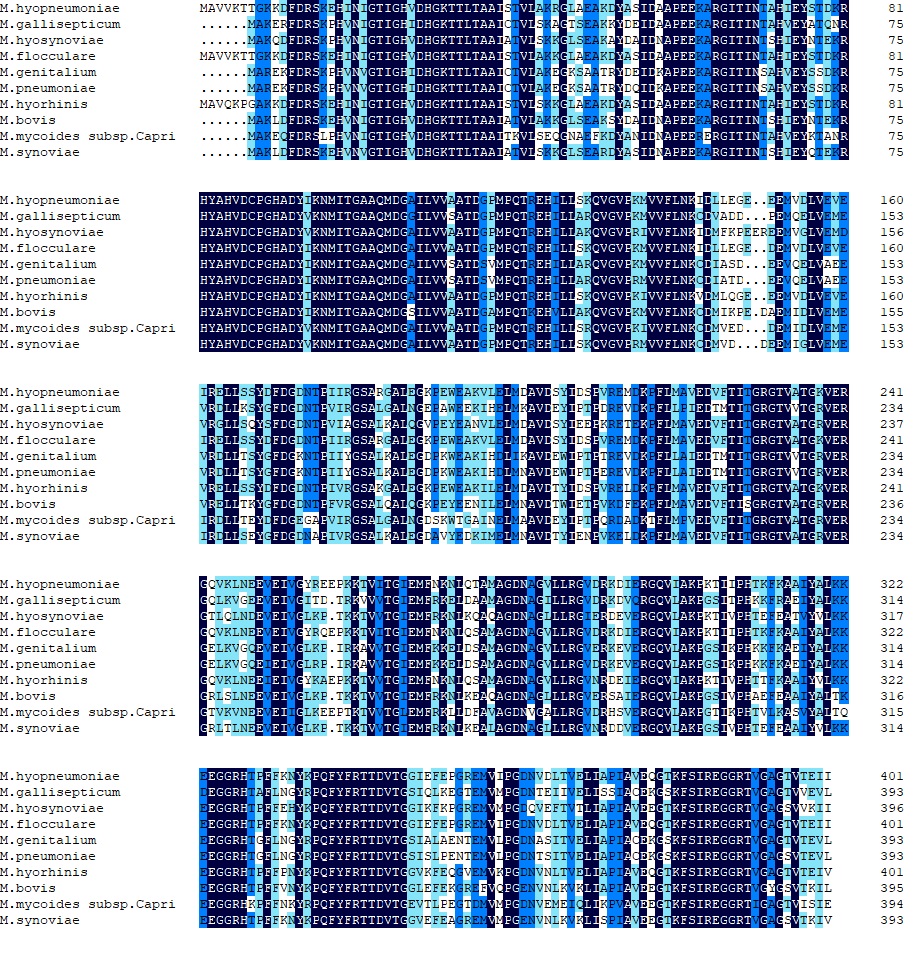

Supplement: Supplemental Material [file KVIR_A_1806664_SM6441.jpg]
